# Alexithymia and attachment dimensions in relation to parental burnout: A structural equation modelling approach

**DOI:** 10.1371/journal.pone.0334647

**Published:** 2025-11-05

**Authors:** Dawid Konrad Ścigała, Joanna Sikora-Ścigała, Elżbieta Zdankiewicz-Ścigała

**Affiliations:** 1 Institute of Psychology, The Maria Grzegorzewska University in Warsaw, Poland; 2 Institute of Psychology, The Maria Grzegorzewska University in Warsaw, Poland; 3 Faculty of Psychology, University of Social Sciences and Humanities, Poland; Wyższa Szkoła Kształcenia Zawodowego: University College of Professional Education, POLAND

## Abstract

**Background:**

Parental burnout is a chronic, parenting-specific syndrome marked by emotional exhaustion, emotional distancing from one’s children, and reduced parental fulfilment. Although links of insecure attachment and emotion-processing difficulties with parental burnout have been reported, their joint associations remain underexplored. This study applied Structural Equation Modelling (SEM) to examine whether alexithymia—defined as difficulties identifying and describing feelings and externally oriented thinking, assessed with the Toronto Alexithymia Scale (TAS-20)—is involved in the associations between attachment orientations and parental burnout, and whether these associations differ by sex.

**Methods:**

A cross-sectional sample of 440 Polish parents (229 women, 211 men; 52.1% women; M = 38.91, SD = 7.33; age range = 21−61) completed the Experiences in Close Relationships—Relationship Structures Questionnaire (ECR-RS), the Toronto Alexithymia Scale (TAS-20), and the Parental Burnout Assessment (PBA). Sex-stratified SEMs were estimated.

**Results:**

In women, higher avoidance toward the mother was directly associated with higher burnout, whereas anxiety toward the mother related to burnout indirectly via elevated alexithymia. In men, avoidance of the mother was directly associated with burnout, while anxiety toward the mother related to burnout indirectly through alexithymia. Parallel patterns emerged for paternal attachment in sex-specific models.

**Conclusions:**

Across sex-stratified models, alexithymia was consistently implicated in the associations between insecure attachment and parental burnout. The patterns differed for women and men, underscoring the value of emotion-focused and attachment-informed support tailored by sex. Findings reflect cross-sectional associations and do not imply causality.

## 1. Introduction

Parental burnout has been increasingly recognized as a significant psychological concern, distinct from general stress [1]. It arises from chronic strain within the caregiving role, often fuelled by emotional overload, insufficient recovery periods, and perceived inadequacy in fulfilling parental responsibilities [2,3]. Parental burnout has a direct impact on the parent–child relationship, often leading to emotional distancing and a diminished sense of parental competence. While socio-cultural and economic factors certainly shape parenting stress, research findings consistently indicate that psychological processes—particularly difficulties in understanding and regulating emotions—are more closely associated with parental burnout than external socio-economic conditions, such as financial difficulties or cultural norms [4]. Parental burnout typically manifests through emotional exhaustion, a sense of detachment from one’s children, and a perceived loss of parental fulfilment [1,5,6].

One specific dimension of emotional processing that has been examined less frequently in the context of parental burnout is alexithymia, defined as a trait-like tendency to experience difficulties identifying and describing emotions, combined with a tendency toward externally oriented thinking [7]. Recent research further indicates that elevated levels of alexithymia are associated with parental burnout, with self-efficacy and psychological resilience potentially mediating this association [8]. Given that parenting requires continuous adjustment to one’s own emotional states as well as to the child’s needs, alexithymia may impair stress regulation and thereby increase vulnerability to parental burnout. In addition, research shows that alexithymia relates to parental burnout by disrupting emotion regulation and reducing resilience to stress [8,9]. Emotion dysregulation associated with alexithymia can exacerbate parenting stress, particularly in circumstances that require high levels of caregiving involvement [10,11]. The present study, therefore, examines whether alexithymia is involved in the association between insecure attachment orientations and parental burnout, providing a more comprehensive account of the psychological processes that may underlie this condition. In this context, alexithymia is operationalized through its established components—difficulties identifying and describing feelings (TAS-20 DIF/DDF) and externally oriented thinking (TAS-20 EOT)—rather than broad terms such as “emotional self-awareness.” These emotional processing limitations have been linked to reduced empathy and impaired social cognition, which may further intensify relational strain in the parent–child relationship [12,13]. By affecting both intra- and interpersonal emotion regulation, alexithymia may represent one of the plausible pathways through which insecure attachment orientations are associated with parental burnout.

### 1.1 Attachment dimensions and emotion regulation deficits

Attachment theory provides a framework for understanding individual differences in emotion regulation and interpersonal functioning [13]. According to this perspective, early caregiver interactions shape internal working models of relationships, influencing emotion regulation, stress adaptation, and coping strategies across the lifespan [14]. Individuals with a secure attachment orientation typically develop more adaptive regulation strategies, which support resilience to stress. In contrast, individuals with insecure orientations—whether anxious or avoidant—tend to rely on less effective regulation strategies, which may be linked to greater difficulties under chronic parental stress [15].

Those high in attachment avoidance often suppress emotions, minimize reliance on others, and disengage from emotionally demanding interactions. Such patterns of emotional distancing are associated with accumulated stress and challenges in adaptive regulation [15,16]. Studies suggest that avoidantly oriented parents may overlook early signs of emotional strain, resulting in persistent suppression and heightened parental exhaustion [17,18]. Conversely, individuals with high attachment anxiety frequently show heightened activation of the attachment system, accompanied by excessive reactivity, fear of inadequacy, and ruminative focus on parenting challenges. These tendencies have been associated with emotional exhaustion and diminished parental confidence [19,20]. In addition, maladaptive cognitive dimensions—such as rumination or catastrophic interpretations of parental difficulties—have been shown to mediate the links between attachment anxiety and burnout [21].

Within this framework, insecure attachment, particularly when combined with difficulties in emotional processing, appears closely related to parental burnout. Alexithymia—conceptualized as a trait-like tendency to experience the challenges of identifying and describing feelings, and to adopt an externally oriented thinking style—has frequently been linked to adverse attachment histories and limited development of adaptive regulation strategies [7,22,23]. Empirical work indicates that both anxious and avoidant orientations are associated with higher levels of alexithymia, suggesting that deficits in labelling and expressing emotions may be one of the plausible pathways connecting insecure attachment with parental burnout.

While earlier studies have examined insecure attachment and parental burnout largely as separate constructs, fewer have focused on emotional processing deficits as an explanatory link. The present study, therefore, tests a mediation model in which alexithymia is examined as a potential connecting factor between insecure attachment and parental burnout. By investigating this pathway, the study aims to extend current understanding of how attachment-related emotional characteristics may contribute to the accumulation of parenting-related strain. These considerations remain primarily theoretical, and future research is needed to clarify whether interventions targeting emotional processing could support the reduction of parental burnout.

### 1.2 Alexithymia as a mediator between attachment and parental burnout

Alexithymia is generally regarded as a relatively stable, trait-like characteristic reflecting persistent difficulties in identifying and describing feelings, as well as a tendency toward externally oriented thinking, rather than a transient stress reaction [7]. Research indicates that elevated alexithymia levels are often linked to early insecure attachment experiences and may continue into adulthood, shaping emotion regulation difficulties [22]. Neurobiological studies further support this conceptualization, showing associations between alexithymia and structural or functional variations in brain regions involved in emotional processing [23].

Individuals with elevated alexithymia often struggle to regulate emotions adaptively and may rely on maladaptive coping strategies, including psychoactive substance use, aggressive behaviour, or somatization [24,25]. Alexithymia has been associated with greater hostility and externalized aggression, particularly in individuals prone to emotion dysregulation [26], as well as with psychosomatic complaints and a reliance on somatization as a defensive strategy [27].

Such difficulties in emotion processing may intensify parenting-related strain by limiting the ability to recognize distress, to respond flexibly to children’s emotions, and to employ constructive coping strategies. Parents with elevated alexithymia may:

experience difficulties recognizing early signs of emotional distress, leading to greater stress accumulation, show limited attunement to their children’s emotional states, resulting in detachment and diminished parental satisfaction, and possess fewer adaptive coping strategies, increasing the likelihood of emotional exhaustion.

Given the established links between insecure attachment orientations and higher alexithymia, and between alexithymia and emotion regulation deficits, alexithymia can be considered one plausible pathway through which attachment insecurity relates to parental burnout. This proposed mediating role directly reflects the theoretical model tested here using Structural Equation Modelling (SEM). Most studies in this area have focused primarily on mothers, with comparatively little attention given to fathers. However, emerging evidence suggests that paternal alexithymia may operate differently and thus warrants separate analysis [28]. Moreover, relatively few studies have examined the specific indirect pathways—such as the role of social support or coping strategies—through which alexithymia may relate to parental burnout [29]. Finally, little research has considered whether the strength of the alexithymia–burnout association varies by attachment orientation or by the quality of early caregiver relationships [30].

### 1.3 Sex-based differences in the pathways to parental burnout

We anticipated that the indirect association of anxious attachment with parental burnout, via alexithymia, would be more pronounced in men, while in women, differences in emotional reactivity might be involved through alternative pathways. Prior research suggests that sex differences in emotion regulation associated with attachment orientations may shape stress management and vulnerability to parental burnout. In the literature, men are often described as more likely to rely on avoidance- and suppression-based strategies, potentially reducing emotional engagement in parenting and relating to higher burnout levels [4,31,32]. Women are more frequently described as using emotion-focused strategies, which—although adaptive in some contexts—may contribute to heightened emotional sensitivity and stress reactivity when parenting demands are chronic [33,34]. In the present study, these patterns are considered as theoretical background rather than directly measured constructs.

In line with this framework, the current study examines whether attachment insecurity and alexithymia show sex-specific associations with parental burnout. Specifically, we expected that:

- Among men, anxious attachment to both mother and father would be indirectly associated with burnout via alexithymia, while avoidant attachment would show a direct association.
- Among women, avoidant attachment to the mother would be directly associated with higher burnout levels, whereas anxious attachment would be indirectly associated with burnout through alexithymia.

These expectations reflect the proposed mediation model and provide the rationale for the sex-stratified analyses reported below.

### 1.4 Study contribution and hypotheses

By examining sex-specific mechanisms associated with parental burnout, this study aims to identify distinct pathways linking attachment orientations, alexithymia, and parental burnout in women and men. Our approach integrates attachment theory, research on alexithymia, and parental burnout frameworks, providing insights into how difficulties in emotion regulation and maladaptive stress responses may be involved in this phenomenon. While previous research has examined the associations between insecure attachment and emotion regulation [20], as well as between alexithymia and psychological maladaptation [7,35,36], no prior study has tested alexithymia as a potential mediator of the attachment–burnout association using Structural Equation Modelling (SEM).

Building on prior findings regarding sex differences in emotion regulation and stress responses, we expected that avoidant attachment would show stronger associations with parental burnout in men, whereas anxious attachment would show indirect associations with burnout via alexithymia in women. These expectations are consistent with literature suggesting that men are more likely to engage in avoidance-based responses that may reduce emotional engagement in parenting, while women may exhibit higher emotional reactivity, which can be linked with greater strain under chronic demands [37,38]. In the present study, these coping-related patterns are treated as theoretical background rather than as directly measured constructs.

By combining neurobiological and psychosocial perspectives, this study examines how early emotional experiences may be reflected in long-term emotion regulation tendencies and vulnerabilities in the parenting role. Furthermore, the sex-stratified analyses clarify psychological processes potentially linked to parental exhaustion. The findings are intended to provide empirical evidence with practical implications, including the potential for tailoring interventions to parents who present an elevated risk of burnout.

#### 1.4.1 General hypothesis.

H1. Insecure attachment to parents is expected to be associated with higher levels of parental burnout. Avoidant attachment was anticipated to show a stronger direct association, whereas anxious attachment was expected to relate to parental burnout indirectly through elevated alexithymia.

Sex-Based Hypotheses

H2. Among men, avoidant attachment to the mother was expected to show the strongest direct association with parental burnout. Both avoidant and anxious attachment were expected to be significantly associated with elevated alexithymia, consistent with the role of emotional suppression in stress regulation.

H3. Among women, avoidant attachment to the mother was expected to show a stronger association with parental burnout than avoidant attachment to the father. Anxious attachment to both parents was expected to be associated with elevated alexithymia, which in turn would be linked to parental burnout.

Mediating Role of Alexithymia

H4. Elevated alexithymia was expected to serve as a mediating factor in the associations between insecure attachment and parental burnout, with anxious attachment showing a stronger indirect association than avoidant attachment.

H5. Given that anxious attachment in men has been linked to greater difficulties in emotion regulation, it was expected to function as a stronger indirect pathway to parental burnout via alexithymia in men than in women.

## 2. Materials and methods

### 2.1. Participants and procedure

The study was conducted in accordance with the recommendations of the Research Ethics Committee of the Maria Grzegorzewska University in Warsaw (Consent No. 235–2019/2020). All procedures involving human participants complied with the institutional research committee’s standards, the Declaration of Helsinki (1964, as amended), and relevant national regulations. Participants were recruited through social media platforms and announcements distributed in educational and healthcare institutions. Before participation, all individuals received detailed written information about the study’s purpose, procedures, and their rights. Each participant provided informed consent before completing the questionnaires. No financial or material compensation was offered for participation.

Data collection was conducted individually and in person in Poland between June 2020 and October 2022. Participants who did not complete all questionnaires or did not meet inclusion criteria (having a typically developing child) were excluded from the analyses. The final sample comprised 440 parents (229 women, 211 men; 52.1% women) of children with typical neurodevelopment (M child age = 9.10 years, SD = 5.68). The mean age of participants was 38.91 years (SD = 7.33; range = 21–61). For women, the mean age was 37.98 years (SD = 6.63; range = 21–56), while for men it was 39.91 years (SD = 7.92; range = 23–61).

Regarding relationship status, 327 participants (74.3%) were married, 73 (16.6%) were in a committed partnership, and 40 (9.1%) were single parents. In terms of education level, 58.4% had higher education, 35.2% had secondary education, and 6.4% had vocational education.

An a priori power consideration indicated that the available sample size (N = 440) exceeded common recommendations for structural equation modelling with multiple latent constructs and indirect pathways. Methodological guidelines suggest that samples above 200 provide adequate power for detecting small-to-moderate effects in SEM [39–41]. Our sample, therefore, provided sufficient precision for the models tested..

### 2.2. Measures

#### 2.2.1. Parental Burnout Assessment (PBA).

Parental Burnout Assessment (PBA).

Parental burnout was assessed using the Polish adaptation of the Parental Burnout Assessment (PBA) [1], developed by Szczygieł et al. [42]. The questionnaire consists of 23 items covering four dimensions: exhaustion (9 items; e.g., “I feel completely run down by my role as a parent”), contrast (6 items; e.g., “I don’t think I’m the good father/mother that I used to be to my child(ren)”), feelings of being fed up (5 items; e.g., “I can’t stand my role as father/mother anymore”), and emotional distancing (3 items; e.g., “I do what I’m supposed to do for my child(ren), but nothing more”). Responses were provided on a 7-point Likert scale ranging from 0 (Never) to 6 (Every day).

The Polish adaptation of the PBA has demonstrated high reliability in previous studies. In the present sample, internal consistency was also high for the total score and subscales (overall α = .96; exhaustion α = .93; contrast α = .90; fed up α = .89; emotional distancing α = .72). When calculated separately, Cronbach’s α values were acceptable in both the female and male subsamples (see S1 Table).

#### 2.2.2. Toronto Alexithymia Scale (TAS-20).

Toronto Alexithymia Scale (TAS-20).

Alexithymia was assessed using the Polish adaptation of the Toronto Alexithymia Scale (TAS-20) [43], validated in its Polish adaptation by Ścigała et al. [44]. The TAS-20 comprises 20 items measuring three dimensions: difficulty identifying feelings (DIF), difficulty describing feelings (DDF), and externally oriented thinking (EOT). Responses are recorded on a 5-point Likert scale ranging from 1 (Strongly disagree) to 5 (Strongly agree), with total scores ranging from 20 to 100. Scores ≥ 61 indicate elevated alexithymia, 52–60 suggest possible (borderline) alexithymia, and ≤ 51 indicate low alexithymia [43,44].

In the present sample, the TAS-20 demonstrated acceptable reliability (total α = .82; DDF α = .75; DIF α = .64; EOT α = .57), consistent with prior validations [43]. Cronbach’s α values were also calculated separately for women and men, confirming comparable reliability across subsamples (see S1 Table).

#### 2.2.3. Experiences in Close Relationships – Relationship Structures (ECR-RS).

Experiences in Close Relationships – Relationship Structures (ECR-RS).

Attachment dimensions were assessed using the Polish adaptation of the Experiences in Close Relationships – Relationship Structures questionnaire (ECR-RS) [42], adapted by Lubiewska et al. [45]. The ECR-RS assesses attachment-related anxiety and avoidance toward four key figures: mother, father, romantic partner, and close friend. In the present study, only the mother and father subscales were analyzed. The questionnaire consists of 36 items (9 per attachment figure), with three items measuring avoidance and six items measuring anxiety. Responses are given on a 7-point Likert scale ranging from 1 (Strongly disagree) to 7 (Strongly agree). Example items include: “I can easily rely on this person” and “I prefer not to show this person how I feel.”

In the present sample, the Polish adaptation demonstrated good internal consistency: avoidance α = .86 (mother) and α = .87 (father); anxiety α = .85 (mother) and α = .86 (father). Cronbach’s α values were also calculated separately for women and men, and confirmed comparable reliability across subsamples (see S1 Table).

### 2.3. Data analysis

Statistical analyses were conducted using IBM SPSS Statistics 28 and AMOS 24. The maximum likelihood (ML) estimator was used in all SEM analyses. Before testing the hypothesized relationships, preliminary analyses were conducted to examine the distributions of variables, assess normality, and detect potential outliers. Additionally, independent samples t-tests were performed to investigate sex differences in attachment dimensions, alexithymia, and parental burnout. The effect size for group comparisons was estimated using Cohen’s d. According to conventional benchmarks, values of approximately.2,.5, and.8 were interpreted as indicating small, medium, and large effects, respectively. To test the proposed relationships, Structural Equation Modelling (SEM) was performed. Model fit was evaluated using several fit indices, including the chi-square statistic (χ²/df), where values ≤ 5 indicate an acceptable model fit, and the Root Mean Square Error of Approximation (RMSEA), with values ≤ .05 indicating a close fit and values between.05 and.08 **c**onsidered acceptable. The Comparative Fit Index (CFI) and Tucker-Lewis Index (TLI) were also used, with values ≥ .95 indicating a good fit. These indices are particularly robust in smaller samples, as noted in prior literature [39–41]. To compare and select models, Akaike’s information criterion (AIC) and Bayesian information criterion (BIC) were utilized, prioritizing models with lower values due to their greater parsimony and ease of interpretation [40]. Finally, Hoelter’s critical N was calculated to determine the minimum sample size required for a reliable model fit, ensuring that the results were not unduly influenced by sampling variability [40].

Following a theory-constrained model-trimming approach [40,41], paths that were statistically non-significant and not theoretically central were removed to improve parsimony and overall fit. This procedure is consistent with established SEM practices, where alternative models are compared on both theoretical and empirical grounds. Competing models and their fit indices are reported, and the final models represent the best balance of statistical adequacy and theoretical coherence.

## 3. Results

The first step in the statistical analysis was to examine differences between male and female respondents in attachment, alexithymia, and parental burnout. To test specific aspects of the study hypotheses (H1–H3), independent-samples t tests were used. Men reported higher levels of anxiety in attachment relationships with both mother and father; however, the difference reached statistical significance only for anxiety toward the mother (Table 1). Women demonstrated significantly higher levels of avoidance in relationships with their fathers, while men showed higher, though non-significant, avoidance toward their mothers.

**Table 1:** Descriptive statistics and sex comparison using independent t tests.

	Female		Male				
VARIABLE	M	SD	M	SD	*t*	*p*	*d cohen*
ECR-R Mother Avoidance	2.85	1.41	2.96	1.51	*0.77*	*0.444*	*0.28*
ECR-R Mother Anxiety	1.92	1.42	2.27	1.54	*2.32*	*<.05*	*0.44*
ECR-R Father Avoidance	3.46	1.58	3.1	1.37	*2.65*	*<.05*	*0.07*
ECR-R Father Anxiety	2.11	1.45	2.21	1.49	*0.69*	*0.487*	*0.27*
Parental burnout PBA Total	21	17.38	29.26	24.48	*3.86*	*<.001*	*0.39*
PBA Exhaustion	12.02	9.89	17.13	14.41	*4.23*	*<.001*	*0.42*
PBA Contrast	4.56	4.49	7.21	7.55	*4.33*	*<.001*	*0.43*
PBA Saturation	3.39	3.88	5.34	5.79	*4.02*	*<.001*	*0.40*
PBA Distancing	1.82	2.08	3.97	3.98	*6.85*	*<.001*	*0.68*
Alexithymia TAS Total	44.11	11.66	50.45	12.12	*5.58*	*<.001*	*0.53*
TAS Difficulty identifying feelings	15.04	5.86	16.29	5.93	*2.23*	*<.05*	*0.21*
TAS Difficulty describing feelings	11.72	3.93	14.06	4.38	*5.89*	*<.001*	*0.56*
TAS Externally oriented thinking	17.35	4.27	20.1	4.51	*6.66*	*<.001*	*0.63*

Note. Cohen’s d values are reported to two decimal places. Effect size interpretation follows conventional guidelines (small = 0.20, medium = 0.50, large = 0.80).

Men also exhibited significantly higher levels of alexithymia compared to women (Table 1). The mean alexithymia score for men (M = 50.45) was close to the conventional cut-off score for elevated alexithymia (≥ 51 points). In addition, men reported significantly higher scores across all subdimensions of parental burnout, as well as higher overall burnout levels (Table 1).

### 3.1. Model testing: the role of attachment and alexithymia in parental burnout

Four Structural Equation Models (SEMs) were estimated to examine the associations of attachment dimensions, alexithymia, and parental burnout separately for women and men. The initial models included all hypothesized paths. To improve overall model fit and parsimony, non-significant paths that were not central to the theoretical framework were removed, following established SEM trimming procedures [40,41]. Revised models and fit indices are reported in detail in the tables below.

#### 3.1.1. Women: relationship between maternal attachment, alexithymia, and burnout.

**Fig 1:**
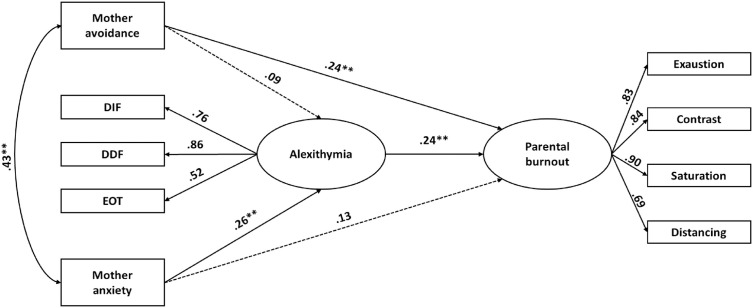
Standardized estimates for the best-fitting model for women, including mother-related avoidance and anxiety attachment dimensions. Dashed lines indicate non-significant paths retained from the theoretical model. Note. p < .05; p < .001.

The first Structural Equation Model (SEM) tested the associations between maternal attachment, alexithymia, and parental burnout among women (Tables 2-3; Fig 1). The initial model included all hypothesized direct and indirect paths. A revised model was subsequently tested by removing statistically non-significant pathways, which resulted in improved fit indices (Table 2).

**Table 2:** Goodness-of-fit indices and model selection indices for females and males in relation to the mother.

Model	χ^2^	df	χ^2^/df	RMSEA	CFI	TLI	AIC	BIC	HOELTER
Female									
Reference model	53.05	23	2.307	.076 (.049,.103)	0.959	0.921	115.05	117.907	151
Best-fitted model	56.316	25	2.253	.074(.048,.100)	0.958	0.924	114.316	116.988	152
Male									
Reference model	29.322	23	1.275	0.036(.000,.071)	0.994	0.987	91.322	94.437	251
Best-fitted model	30.814	24	1.284	0.037(.000,.071)	0.992	0.987	90.814	93.829	247

Note. RMSEA = Root Mean Square Error of Approximation; CFI = Comparative Fit Index; TLI = Tucker–Lewis Index; AIC = Akaike Information Criterion; BIC = Bayesian Information Criterion; HOELTER = minimum sample size required for reliable model fit.

**Table 3:** Standardized estimates and 95% confidence intervals for females in relation to mothers.

Parameter		estimate	95% LLCI	95% ULCI	p value
Female					
Mother avoidance	Parental burnout	0.24	0.080	0.446	<.001
Mother avoidance	Alexithymia	0.09	−0.102	0.212	0.273
Mother anxiety	Parental burnout	0.13	−0.001	0.295	0.052
Mother anxiety	Alexithymia	0.26	0.014	0.342	<.001
Alexithymia	Parental burnout	0.24	0.024	0.354	<.001

Estimate standardized estimates; 95% LLCI, 95% ULCI confidence intervals; p value

In the final model, higher avoidance in the relationship with the mother was directly associated with parental burnout (β = .24, p < .05). Anxious attachment to the mother was not directly associated with parental burnout but was positively associated with alexithymia (β = .26, p < .05). Alexithymia, in turn, was significantly associated with parental burnout (β = .24, p < .001). Avoidance in the relationship with the mother was not significantly associated with alexithymia (β = .09, p = .27).

#### 3.1.2. Men: relationship between maternal attachment, alexithymia, and burnout.

**Fig 2:**
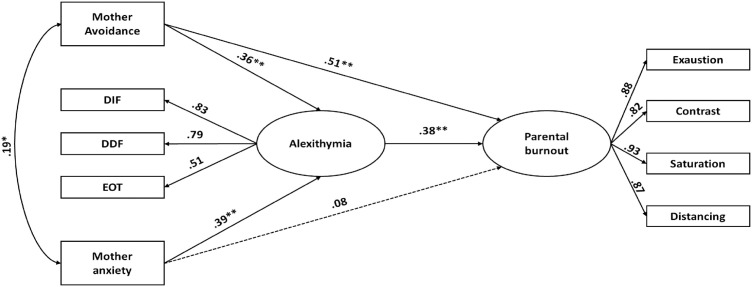
Standardized estimates for the best-fitting model for men, including mother-related avoidance and anxiety attachment dimensions. Dashed lines indicate non-significant paths retained from the theoretical model. Note. p < .05; p < .001.

The second Structural Equation Model (SEM) tested the associations between maternal attachment, alexithymia, and parental burnout among men (Tables 2 and 4; Fig 2). As in the women’s model, two versions of the model were estimated. The initial model included all hypothesized paths, including direct effects of both anxious and avoidant attachment to the mother on parental burnout, as well as indirect effects via alexithymia. The revised model removed the non-significant path from anxious attachment to parental burnout, which improved overall model fit.

**Table 4:** Standardized estimates and 95% confidence intervals for males to the mothers.

Parameter		estimate	95% LLCI	95% ULCI	p value
Male					
Mother avoidance	Parental burnout	0.51	.329	.599	<.001
Mother avoidance	Alexithymia	0.36	.161	.436	<.001
Mother anxiety	Parental burnout	0.08	−.241	.050	0.182
Mother anxiety	Alexithymia	0.39	.249	.537	<.001
Alexithymia	Parental burnout	0.38	.235	.497	<.001

Note. Est. = standardized estimates; 95% LLCI = lower limit of the 95% confidence interval; 95% ULCI = upper limit of the 95% confidence interval; p = significance level.

In the final model, avoidant attachment to the mother was directly associated with parental burnout (β = .30, p < .001). Anxious attachment to the mother was not directly associated with parental burnout but was significantly associated with alexithymia (β = .29, p < .01). In turn, alexithymia was positively associated with parental burnout (β = .22, p < .01).

#### 3.1.3. Women: relationship between paternal attachment, alexithymia, and burnout.

**Fig 3:**
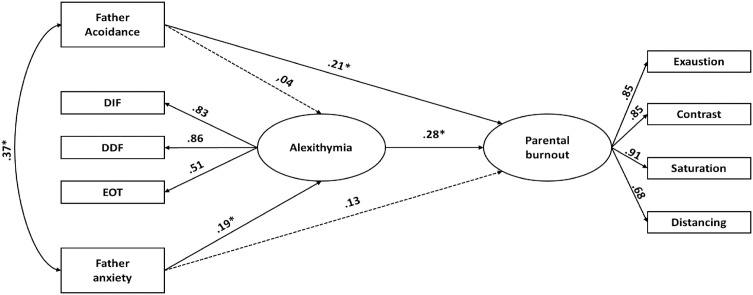
Standardized estimates for the best-fitting model for women, including father-related avoidance and anxiety attachment dimensions. Dashed lines indicate non-significant paths retained from the theoretical model. Note. p < .05; p < .001.

The third Structural Equation Model (SEM) tested the associations between paternal attachment, alexithymia, and parental burnout among women (Tables 5 and 6; Fig 3). As with the previous models, two versions were compared. After the removal of two non-significant paths, the revised model showed better fit indices compared to the original specification.

**Table 5:** Goodness-of-fit indices and model selection indices for females and males to father.

Model	χ^2^	df	χ^2^/df	RMSEA	CFI	TLI	AIC	BIC	HOELTER
WOMEN									
Reference model	46.968	23	2.042	.068 (.039,.095)	0.966	0.934	108.968	111.825	170
Best-fitted model	49.434	25	1.977	.066(.038,.092)	0.966	0.938	107.434	110.107	173
MEN									
Reference model	34.182	23	1.486	0.048(.000,.080)	0.988	0.977	96.182	99.297	216
Best-fitted model	36.836	24	1.535	0.051(.007,.082)	0.987	0.975	96.836	99.851	207

Note. RMSEA = Root Mean Square Error of Approximation; CFI = Comparative Fit Index; TLI = Tucker–Lewis Index; AIC = Akaike Information Criterion; BIC = Bayesian Information Criterion; HOELTER = minimum sample size required for reliable model fit.

**Table 6:** Standardized estimates and 95% confidence intervals for females in relation to the father.

Parameter		estimate	95% LLCI	95% ULCI	p value
Female					
Father avoidance	Parental burnout	0.21	0.024	0.337	0.05
Father avoidance	Alexithymia	0.04	−0.084	0.204	0.613
Father anxiety	Parental burnout	0.13	−0.100	0.202	0.101
Father anxiety	Alexithymia	0.19	0.050	0.260	0.05
Alexithymia	Parental burnout	0.28	0.051	0.391	<0.001

Note. Est. = standardized estimates; 95% LLCI = lower limit of the 95% confidence interval; 95% ULCI = upper limit of the 95% confidence interval; p = significance level.

In the final model, paternal attachment avoidance was significantly associated with parental burnout (β = .21, p = .05). Paternal attachment anxiety was not directly associated with parental burnout (β = .13, p = .10), but it was positively associated with alexithymia (β = .19, p < .05). Paternal attachment avoidance was not significantly associated with alexithymia (β = .04, p = .61). Finally, alexithymia was positively associated with parental burnout (β = .28, p < .001).

#### 3.1.4. Men: relationship between paternal attachment, alexithymia, and burnout.

**Fig 4:**
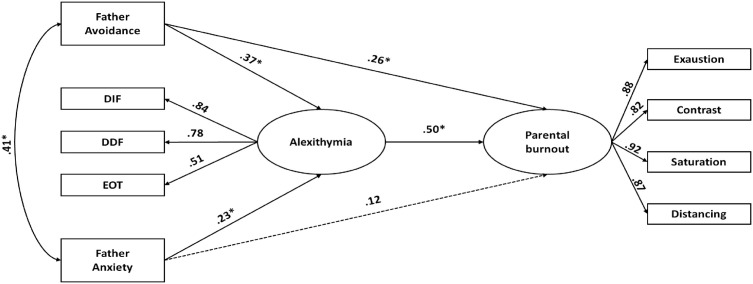
Standardized estimates for the best-fitting model for men, including father-related avoidance and anxiety attachment dimensions. Dashed lines indicate non-significant paths retained from the theoretical model. Note. p < .05; p < .001.

The fourth Structural Equation Model (SEM) tested the associations between paternal attachment, alexithymia, and parental burnout among men (Tables 5–7; Fig 4). As in the maternal models, the revised version provided a better fit than the original. Specifically, the path from paternal attachment anxiety to parental burnout was removed, as it was non-significant.

In the final model, paternal attachment avoidance was significantly associated with parental burnout (β = .26, p < .001). Both paternal attachment avoidance (β = .37, p < .001) and paternal attachment anxiety (β = .23, p < .05) were significantly associated with alexithymia. In turn, alexithymia was positively associated with parental burnout (β = .50, p < .001).

Sex was assessed through a binary self-report format (male/female). This classification reflects a biological sex-based framework and does not encompass non-binary or gender-diverse identities. This limitation should be taken into account when interpreting the findings..

**Table 7:** Standardized estimates and 95% confidence intervals for males to father.

Parameter		estimate	95% LLCI	95% ULCI	p value
Male					
Father avoidance	Parental burnout	0.26	.095	.389	<0.001
Father avoidance	Alexithymia	0.37	.172	.467	<0.001
Father anxiety	Parental burnout	0.12	−.071	.253	0.069
Father anxiety	Alexithymia	0.23	.044	.381	0.05
Alexithymia	Parental burnout	0.5	.306	.587	<0.001

Given well-documented sex differences in emotion regulation strategies and stress responses, separate Structural Equation Models were tested for men and women to examine sex-specific pathways. Prior research has highlighted that attachment dynamics and emotional processing often differ by sex, justifying stratified modelling. Structural Equation Models were estimated for men and women to examine sex-specific pathways. Prior research has documented that attachment dynamics and emotional processing often vary by sex, which provides justification for stratified modelling in the present analyses.

## 4. Discussion

### 4.1. Sex differences in pathways towards parental burnout

The results confirm the presence of sex differences in pathways associated with parental burnout, consistent with previous models of stress regulation and emotion processing. In the male group, avoidant attachment to the mother showed the strongest direct association with burnout. This pattern should be interpreted in relative terms, as both avoidant and anxious attachment dimensions were included in the model; thus, the finding reflects the comparatively stronger statistical weight of avoidance within the same analysis. Avoidant attachment—characterized in the literature by emotional distancing and reluctance to seek support—has been described as associated with cumulative stress and emotional disengagement in the parental role [46].

Among women, anxious attachment to parents was indirectly associated with parental burnout through elevated alexithymia. Although our data did not include direct measures of emotional reactivity or caregiving involvement, previous studies have linked anxious attachment with greater emotional sensitivity and a tendency to over-engage in caregiving roles [20]. In the present study, such behavioral tendencies are discussed only as possible interpretations suggested by the literature, not as observed variables. These processes may help explain greater vulnerability to emotional exhaustion, a central dimension of burnout. Our findings are also consistent with Ścigała et al. [8], who reported that elevated alexithymia is associated with impaired stress regulation and reduced access to cognitive reinterpretation strategies.

Sex differences in the observed patterns may also be understood in the light of social and cultural factors described in the literature. Research indicates that parents often transmit sex-related emotional roles to children, shaping later stress regulation and caregiving behaviors [47,48]. Cross-cultural studies have shown that in collectivist societies, women are more often socialized into emotion-focused coping strategies, whereas men are encouraged to rely on suppression and avoidance [49]. While such interpretations are based on previous research rather than direct measures in this study, they may help contextualize why anxious attachment appeared more prominent among women, whereas avoidant attachment was more salient among men, in association with burnout [50,51].

Finally, data collection took place during the COVID-19 pandemic—a period marked by heightened parental stress, reduced social support, and disrupted routines. These contextual conditions may have amplified burnout levels and should be considered when interpreting the findings. A statement reflecting this limitation has been included in the manuscript.

### 4.2. The role of alexithymia as a mediator

In all models, elevated alexithymia emerged as a consistent mediating factor, supporting the hypothesized role of emotion-processing difficulties in parental burnout. In the female group, alexithymia was primarily associated with anxious attachment, suggesting that greater emotional insecurity in parental relationships may be linked to difficulties in identifying and expressing emotions, which in turn relate to higher exhaustion. These results are in line with neurobiological findings showing that elevated alexithymia is associated with reduced activity in brain regions responsible for affective processing [23].

In the male group, both attachment avoidance and attachment anxiety were significantly associated with elevated alexithymia, indicating that different forms of insecure attachment may be linked to difficulties in emotional awareness and regulation in this context. Elevated alexithymia consistently showed significant associations with parental burnout across models, suggesting that it may represent an important vulnerability factor rather than only a stress reaction.

Sex differences described in the literature may further help to interpret these patterns. Previous research indicates that men are more likely to report difficulties in identifying emotions and to rely on suppression strategies, whereas women tend to show greater awareness of affective states but more challenges in constructive expression [52,53]. Although not directly measured in this study, such tendencies could mean that men with elevated alexithymia may be particularly affected by combined difficulties in emotion recognition and regulation, while in women, higher burnout levels may be linked to emotional over-reactivity and difficulties in constructive expression.

It is also noteworthy that in our sample, men exhibited significantly higher mean alexithymia scores than women, a pattern consistent with previous research documenting higher average alexithymia in male populations [43,44].

### 4.3. Attachment dimensions vs. risk of burnout

An important contribution of the present study is showing that maternal and paternal attachment may demonstrate differential associations with parental burnout, consistent with attachment theory perspectives on the role of both parents in emotional development. In the male group, avoidant attachment to the mother showed the strongest direct association with burnout. While our data do not directly assess developmental pathways, this pattern is in line with literature identifying the mother as a primary attachment figure and suggesting that early lack of emotional closeness may be associated with long-term challenges in stress regulation [14,15].

In the female group, avoidant attachment to the father was more strongly associated with burnout than avoidant attachment to the mother, underscoring—according to previous studies—the potential importance of the father–daughter bond for resilience to stress and effective emotion regulation. Furthermore, in women, anxious attachment to the father showed a stronger association with elevated alexithymia than anxious attachment to the mother, suggesting that emotional insecurity in the father–daughter relationship may play a distinctive role in emotion processing and its links with burnout. These interpretations are grounded in prior research and not in direct measurement within the present study.

The literature also indicates that attachment to the mother and father may affect emotion regulation and parental burnout in different ways. Avoidant attachment to the father has been associated with reduced stress resilience and greater emotion-processing difficulties in daughters, whereas avoidant attachment to the mother has been more strongly linked to emotional distancing in sons [54,55]. Longitudinal studies further suggest that insecure attachment to either parent may increase vulnerability to emotion dysregulation, which in turn has been associated with higher levels of burnout [56].

In summary, the results reveal sex differences in the associations between attachment and parental burnout. Among men, avoidant attachment to the mother was both directly and indirectly (via elevated alexithymia) associated with burnout, while anxious attachment to the mother showed only an indirect association. Among women, avoidant attachment to the mother was directly associated with burnout, and anxious attachment was indirectly associated via alexithymia. Notably, avoidant attachment to the father showed a stronger association with burnout in women than avoidance toward the mother, highlighting—consistent with prior research—the role of the father–daughter bond in fostering emotional resilience and stress regulation. Furthermore, anxious attachment to the father was more strongly associated with elevated alexithymia than anxious attachment to the mother, suggesting that emotional insecurity in the father–daughter relationship may be particularly relevant to emotion-processing difficulties and parental burnout

## 5. Theoretical implications

The present study contributes to attachment theory by indicating that early-life attachment patterns may persist and be reflected in emotion regulation and stress management in adulthood. By identifying elevated alexithymia as a consistent mediator, the findings align with theoretical frameworks suggesting that difficulties in emotion regulation represent one of the pathways linking insecure attachment with psychological distress [7]. Furthermore, the results refine existing models of parental burnout by suggesting that avoidant attachment is more strongly associated with burnout in men, whereas anxious attachment shows stronger indirect associations in women via alexithymia. These patterns are consistent with literature conceptualizing alexithymia as a relatively stable vulnerability factor for parental burnout, although the cross-sectional design precludes causal inference. Individuals with elevated alexithymia levels consistently report difficulties in emotion regulation, which are associated with reduced capacity to cope effectively with parenting-related stress [9,28,56–58]. This interpretation is further supported by neurobiological evidence linking alexithymia to structural alterations in brain regions involved in emotional processing, such as the anterior cingulate cortex and insula [8,23].

The findings also emphasize that sex differences may be central to the dynamics of parental functioning, shaping the pathways through which early attachment schemas are reflected in parenting relationships [29,30]. Moreover, this study highlights elevated alexithymia as a potential transdiagnostic factor connecting early attachment experiences with parental burnout, pointing to its relevance as a target for preventive interventions across both women and men.

## 6. Practical implications

From a practical perspective, the findings highlight the importance of tailored interventions addressing parental burnout, with a focus on emotion regulation and attachment-related processes. Programs may include emotion regulation training and attachment-informed approaches, particularly for parents with a history of insecure attachment. Such strategies could draw on evidence-based therapeutic methods such as Mentalization-Based Therapy (MBT) or Emotion-Focused Therapy (EFT) to strengthen emotional awareness and interpersonal sensitivity, as well as structured interventions aimed at improving emotion recognition and processing—especially for those presenting with elevated alexithymia.

Given the observed sex differences, sex-sensitive support systems appear warranted. For example, fathers may benefit from interventions designed to foster emotional engagement and reduce reliance on suppression or avoidance, while mothers may benefit from support in managing emotional hyperreactivity and developing constructive forms of expression. These approaches could be delivered through targeted parental counselling, Mindful Parenting programs, group therapy informed by attachment theory, or psychoeducational workshops focused on emotional skills.

Practical applications informed by the present findings may include:

- attachment-based interventions for individuals with insecure attachment histories,
- emotion regulation training (e.g., cognitive reappraisal, mindfulness-based stress reduction) with modules tailored to alexithymic tendencies,
- sex-sensitive parental support programs addressing fathers’ emotional engagement and mothers’ emotion regulation,
- community-based parenting groups that integrate psychoeducation on stress management with peer support.

Integrating such approaches into parental counselling, stress management programs, and psychological services may help to address parental burnout and strengthen family well-being.

Finally, this study assessed sex using a binary, biological framework, which does not capture the experiences of non-binary or gender-diverse parents. Future research should incorporate more inclusive measures to address this limitation.

## 7. Limitations and future directions

Although this study contributes to understanding the associations between insecure attachment, elevated alexithymia, and parental burnout, several limitations should be acknowledged.

First, the cross-sectional design precludes causal inference. Longitudinal studies are needed to examine how attachment, alexithymia, and parental burnout evolve and interact over time.

Second, reliance on self-report measures may have introduced bias due to social desirability or limited self-awareness. Future research should incorporate objective indicators of emotion and stress regulation, such as physiological measures (e.g., heart rate variability, cortisol levels) or behavioural observations.

Third, while our model focused on psychological factors such as attachment orientations and emotion regulation capacities, future studies should examine their interplay with cultural, social, and economic factors to develop a more comprehensive account of parental burnout.

Fourth, coping styles and related behavioural mechanisms discussed in the introduction were not directly measured here and should be addressed in future studies to clarify their potential moderating roles.

Finally, data collection occurred during the COVID-19 pandemic—a period of heightened stress, reduced social support, and altered family routines—which may have amplified reported levels of parental burnout. These contextual conditions should be considered when interpreting the results. Future work would also benefit from including more diverse and representative samples, along with inclusive measures of gender identity, to better reflect the experiences of non-binary and gender-diverse parents.

### 7.1. Conclusion

In summary, this study advances understanding of how insecure attachment orientations are associated with parental burnout through the mediating role of elevated alexithymia, while also revealing distinct pathways for women and men. The findings highlight the importance of considering sex-specific mechanisms when designing interventions aimed at strengthening emotion regulation skills and addressing parental burnout.

To our knowledge, this is the first study to apply Structural Equation Modelling (SEM) to examine alexithymia as a mediator of the attachment–burnout association. The use of SEM enabled the simultaneous estimation of complex, interrelated pathways, providing a more integrated and statistically robust test of the proposed model than would be possible with traditional analytic approaches.

By combining an innovative methodological framework with a theoretically grounded model, this research fills an important gap in the parental burnout literature and offers a template for future studies seeking to integrate multiple psychological constructs within a single, coherent analytical structure.

## Supporting information

S1 Table Cronbach’s alpha reliability coefficients of the instruments, calculated separately for women and men. (DOCX)

S1 Data Research data file. (XLSX)
