# Supplementary material for: Alexithymia and attachment dimensions in relation to parental burnout: A structural equation modelling approach
Source: PLoS One. 2025 Nov 5;20(11):e0334647. doi: 10.1371/journal.pone.0334647 (PMC12588487; doi:10.1371/journal.pone.0334647)
Supplement: S1 Table — (DOCX) [file pone.0334647.s001.docx]

|  | | | Female | Male |
| --- | --- | --- | --- | --- |
| VARIABLE | | |  |  |
| ECR-R Mother Avoidance | | | 0.876 | 0.839 |
| ECR-R Mother Anxiety | | | 0.828 | 0.873 |
| ECR-R Father Avoidance | | | 0.887 | 0.843 |
| ECR-R Father Anxiety | | | 0.829 | 0.943 |
| Parental burnout PBA Total | | | 0.963 | 0.967 |
| PBA Exhaustion | | | 0.927 | 0.942 |
| PBA Contrast | | | 0.898 | 0.908 |
| PBA Saturation | | | 0.892 | 0.893 |
| PBA Distancing | | | 0.742 | 0.722 |
| Alexithymia TAS Total | | | 0.832 | 0.800 |
| TAS Difficulty identifying feelings | | | 0.741 | 0.805 |
| TAS Difficulty describing feelings | | | 0.641 | 0.629 |
| TAS Externally oriented thinking | | | 0.514 | 0.523 |
|  |  |  |  |  |
